# Speech and Swallowing Outcomes Following Surgical Resection with Immediate Free Tissue Transfer Reconstruction for Advanced Osteoradionecrosis of the Mandible Following Radiation Treatment for Head and Neck Cancer

**DOI:** 10.1007/s00455-021-10375-4

**Published:** 2021-10-13

**Authors:** Grainne Brady, Lauren Leigh-Doyle, Francesco Mattia Giovanni Riva, Cyrus Kerawala, Justin Roe

**Affiliations:** 1 Therapies Department, The Royal Marsden NHS Foundation Trust, London, U.K.; 2 Department of Surgery & Cancer, Imperial College London, London, U.K.; 3 Head and Neck Unit, The Royal Marsden NHS Foundation Trust, London, U.K.; 4 Faculty of Health and Wellbeing, University of Winchester, Winchester, U.K.; 5 Department of Otolaryngology, Head and Neck Surgery, Imperial College Healthcare NHS Trust, London, U.K.

**Keywords:** Osteoradionecrosis, Early feeding protocol, Rehabilitation, Functional outcomes

## Abstract

Despite recent advances in the radiation techniques used for the treatment of head and neck cancer (HNC) including intensity-modulated radiotherapy (IMRT), mandibular osteoradionecrosis (ORN) remains a significant complication. Advanced stage ORN is managed surgically with resection and immediate free tissue transfer reconstruction. An evaluation of the functional speech and swallowing outcomes was undertaken for patients undergoing surgical management of advanced ORN. We retrospectively reviewed consecutive patients, at a single, tertiary cancer centre, who underwent surgical resection for advanced Notani grade III ORN. Outcomes investigated included use and duration of tracheostomy and swallowing and speech status using Performance Status Scale for Head and Neck Cancer Normalcy of Diet (PSS-NOD) and Understandability of Speech (PSS-Speech) at baseline and 3 months following surgery. Ten patients underwent surgical resection with free tissue transfer reconstruction between January 2014 and December 2019. Two patients required supplemental nutrition via a gastrostomy at three months post surgery. As per the PSS-NOD data half of the patients’ (*n* = 5) diet remained stable (*n* = 2) or improved (*n* = 3) and half of the participants experienced a decline in diet (*n* = 5). The majority of patients had no speech difficulties at baseline (*n* = 8). The majority of patients’ speech remained stable (*n* = 8) with two patients experiencing a deterioration in speech clarity following surgery. Well-designed studies with robust, sensitive multidimensional dysphagia and communication assessments are required to fully understand the impact of surgical management of advanced ORN using resection with free tissue transfer reconstruction.

## Background

Osteoradionecrosis (ORN) of the mandible is a condition involving chronic non-healing bone infection, leading to mucosal breakdown and permanent bone exposure, which occurs as a late toxicity from previous radiation treatment for head and neck cancer [1]. ORN can cause recurrent infection and pain, pathological fracture and functional decline in terms of both speech and swallowing and has been shown to have a significant impact on quality of life (QoL) [2].

ORN is defined as an area of bone necrosis in a previously irradiated field which fails to heal over a period of 3–6 months without evidence of persisting or recurrent tumour [1]. Various severity classifications are reported in the literature. The Notani classification has been recommended to ensure consistent reporting in clinical trials [3]. It specifies three levels of ORN severity: grade 1 ORN confined to the alveolar bone, grade II ORN limited to the alveolar bone and/ or mandible above the level of the interior alveolar canal and grade III ORN involving the mandible below the level of the inferior alveolar canal and/ or skin fistula and/ or pathological fracture [4].

ORN has been reported to occur across a wide time frame following radiation treatment ranging from a number of months to several years [5, 6]. Risk factors for ORN include patient-related factors such as smoking, poor dental hygiene, alcohol consumption and various comorbidities. As well as tumour involvement of the mandible, treatment-related factors such as the use of 3-dimensional conformal radiation therapy (3D-CRT) as opposed to intensity-modulated radiotherapy (IMRT). In the context of IMRT, prevalence rates of approximately 5% are reported [5–8]. Pre-radiation dental extractions have also been highlighted as a risk factor for later development of ORN [7].

Acute, long-term and late side effects of previous radiation treatment on swallowing function have been well documented in the literature [9–11]. Patients with ORN are at risk of functional decline as a result of the ORN itself [1]. This risk may also be exacerbated by the potential for persisting and late radiation-associated dysphagia (late-RAD) [5, 11]. Previous literature looking specifically at oropharyngeal cancer patients has shown that in patients treated with combined modality intensity-modulated radiotherapy regimens, ORN is associated with a significantly higher prevalence of chronic dysphagia detected by clinical examination (OR 4.6, 95% CI 2.1–10.3) or patient-reported measures [12]. Patients with ORN report various other related difficulties also including trismus, issues with teeth and gums and dry mouth [13, 14].

Surgical interventions for ORN vary based on the degree of damage present, ORN location, and individual patient characteristics with the common goals of relief of persistent pain, infection management, restoration of bone continuity, and functional and QoL improvements. For Notani grade III ORN including involvement of the basal portion of the mandibular bone or the presence of a pathological fracture and/or fistula, mandibulectomy with immediate free tissue transfer reconstruction is often required [1].

Previous literature reporting health-related QoL (HRQoL) following reconstructive surgery for ORN tend to report improved QoL following surgical intervention [15], whereas other studies report long-term issues with HRQoL [2]. These studies are limited by small sample sizes.

Speech and swallowing outcomes have been reported previously. Shan and colleagues report on a series of 5 patients who had bilateral ORN and underwent segmental resection and microvascular fibular flap reconstruction [16]. Improved mouth opening and oral intake of at least soft foods are reported. Baseline level of function and validated swallowing outcome measures are not reported.

Chandarana and colleagues report on the outcomes of 7 patients at 12 months post resection and microvascular fibular flap reconstruction for advanced ORN [17]. Although, a standardised measure of swallowing was not used, reduced gastrostomy use at 12 months post surgery is reported.Baseline speech data were not reported, although at 12 months post treatment, there appeared to be persistent difficulties with speech according to the Performance Status Scale for Head and Neck Cancer Understandability of Speech (PSS-Speech) subtest with a median score was 4 out of 5 (range 2 to 5) [18].

When patients are offered surgical resection and microvascular free flap reconstruction the surgeon and multidisciplinary team should work together with the patient to set appropriate expectations in terms of potential functional and QoL outcomes [2]. Functional outcomes based on validated measures of pre and post swallowing and speech function are not well understood due to a lack of data in the literature.

A single site evaluation of the functional speech and swallowing outcomes was undertaken for patients with Notani grade III ORN including pathological fracture and/or fistula undergoing mandibulectomy with immediate free tissue transfer reconstruction.

## Methods

We retrospectively reviewed consecutive patients, at a single, tertiary cancer centre, who underwent surgical resection management of Notani grade III ORN with free flap reconstruction between January 2014 and December 2019. Data collected included patient demographics, previous disease and treatment, type of flap reconstruction, duration of tracheostomy, length of hospital stay, and Performance Status Scale for Head and Neck Cancer Normalcy of Diet (PSS-NOD) and PSS-Speech scores at baseline and 3 months following surgery (Table 1) [18]. At our centre, an early feeding protocol is implemented in line with current literature for all patients who undergo oral cavity resection with free tissue transfer reconstruction [19].

**Table 1:** Performance Status Scale for Head and Neck Cancer Normalcy of Diet (PSS-HN NOD) and Understandability of Speech (PSS- Speech) subscales

PSS-HN NOD scoring:	PSS-HN Speech scoring:
100 Full diet with no restrictions	100 Always understandable
90 Full diet with liquid assistance	75 Understandable most of the time; occasional repetition necessary
80 All meats	50 Usually understandable; face-to-face
70 Carrots, celery (crunchy)	25 Never understandable; may use written
60 Dry bread & crackers	0 No communication
50 Soft, chewable foods (pasta, canned soft fruits, fish)	
40 Soft foods requiring no chewing e.g. mashed potato, apple sauce	
30 Puree	
20 Warm liquids	
10 Cold liquids	
0 Non oral	

## Results

Ten patients underwent segmental mandibulectomy with immediate free tissue transfer reconstruction for Notani grade III ORN between January 2014 and December 2019. The majority of the sample were male, with a mean age of 68.5 (range 56–81). The mean length of time from definitive treatment for HNC was 8.7 years (rage 5–17 years). Primary disease site included oral cavity (*n* = 5), including anterior tongue (*n* = 3), floor of mouth (*n* = 1) and mandible (*n* = 1), oropharynx (*n* = 4) and unknown primary (*n* = 1). Previous treatment included surgery and adjuvant radiation (*n* = 8) and chemoradiation (*n* = 2). Treatment specifics including previous radiation dosage were not available as the majority of these patients were external referrals for management of ORN having been treated previously elsewhere.

Surgical details including tracheostomy use, surgical resection and reconstruction used and length of hospital stay are summarised in Table 2. The majority of patients had a fibular flap reconstruction (*n* = 8), two patients required a tracheostomy at the time of surgery and were successfully decannulated post surgery. Median time to decannulation was 4 days (range 3–5).

**Table 2:** Surgical details including tracheostomy use, method of reconstruction and length of hospital stay

Flap reconstruction	Anterolateral thigh flap (*n* = 1) Fibular flap (*n* = 8) Deep circumflex iliac artery (DCIA) flap (*n* = 1)
Tracheostomy	Yes: *n* = 2 No: n = 8
Time to decannulation	Median: 4 days (range 3–5)
Length of hospital stay	Median: 9 days (range 7–15)

At our centre, all patients with a diagnosis of ORN are seen for baseline pre-surgical assessment of swallowing function, including instrumental evaluation where possible. In one instance, due to the presence of baseline dysphagia with confirmed silent aspiration with a Penetration- Aspiration Scale (PAS): 8 [20] on videofluoroscopy, a gastrostomy was placed pre-operatively. All patients who do not have a gastrostomy have an NGT placed intraoperatively. One patient required conversion to a gastrostomy following surgery due to increased difficulty swallowing. Again, aspiration was confirmed using videofluoroscopy, (PAS: 8) [20]. An early feeding protocol was used with clinical evaluation of swallowing on day 1 post surgery. All patients were able to tolerate at least oral fluids (sips of water) on initial assessment following surgery. Two patients required supplemental nutrition via a gastrostomy at three months post surgery. There were no flap-related complications (including orocutaneous fistula or total flap failure). Baseline and post-surgical PSS-HN NOD and PSS-HN Speech scores are summarised in Table 3. One patient was managing full oral diet with no restrictions at baseline (*n* = 1), the remaining 9 participants had some level of diet restrictions at baseline (*n* = 9). Half of the patients’ (*n* = 5) diet remained stable (*n* = 2) or improved (*n* = 3) and half of the patients experienced a decline in diet (*n* = 5). The majority of patients had no speech difficulties at baseline. The majority of patient’s speech remained stable (*n* = 8) with two patients experiencing a deterioration in speech clarity.

**Table 3:** Demographics, baseline and post-surgery Performance Status Scale (PSS) for Head and Neck Cancer Normalcy of Diet (PSS-NOD) and Understandability of Speech (PSS-Speech), and gastrostomy status

Participant	Sex	Age (years)	Primary diagnosis	Previous treatment	PSS-NOD Baseline	PSS-NOD 3 months	PSS Speech baseline	PSS Speech 3 months	Tube baseline	Tube 3 months
1	F	69	*SCC oropharynx	Surgery + **PORT	50	100	100	100	No tube	No tube
2	F	66	Mucoepidermoid cancer tongue	Surgery + **PORT	50	30	100	75	No tube	No tube
3	M	64	*SCC oropharynx	Surgery + **PORT	70	50	100	100	No tube	No tube
4	M	72	*SCC floor of mouth	Surgery + **PORT	50	50	75	75	No tube	No tube
5	F	81	*SCC mandible	Surgery + **PORT	100	100	75	75	No tube	No tube
6	M	69	*SCC tongue	Surgery + **PORT	50	30	100	75	No tube	G-Tube
7	M	72	*SCC oropharynx	Surgery + **PORT	50	30	100	100	No tube	No tube
8	M	56	*SCC oropharynx	Chemoradiation	50	90	100	100	No tube	No tube
9	F	65	Unknown primary	Chemoradiation	40	50	100	100	No tube	No tube
10	M	71	*SCC tongue	Surgery + **PORT	40	30	100	100	No tube	G-Tube

*SCC squamous cell carcinoma

**PORT post-operative radiation treatment

## Discussion

To our knowledge, this is the first case series of patients reporting baseline and post-operative validated measures of both speech and swallowing following surgical resection with immediate free tissue transfer reconstruction for Notani grade III ORN.

This study is not without its limitations, representing a single centre experience with a small cohort of only 10 patients. Information regarding previous treatments received by the patients is limited as the majority of these patients were treated for their primary disease external to our institution. Due to the small number of participants, statistical analysis would not be representative. Unidimensional clinician-reported measures of swallowing/ speech function are reported. Data on instrumental evaluation of swallowing function pre and post surgery were inconsistently collected in addition to patient-reported outcome measures and this is not reported. Other pertinent clinical issues such as trismus are not reported.

In keeping with previous studies highlighting the risk of swallowing difficulties in patients with ORN [2, 11], the majority of our cohort of patients were experiencing some form of swallowing difficulty with dietary restrictions at baseline. This may be the result of the ORN itself and/or previous surgery or radiation treatments received.

In contrast to some studies reporting improvements in oral intake following resection and free flap reconstruction for ORN [15, 16], our study demonstrated more mixed results including half of the patients remaining stable or improving, and half of the patients experiencing some deterioration in swallowing function. In our small cohort, increased gastrostomy use was also noted. However, as one of the two patients who required a gastrostomy had this placed pre-operatively, it is impossible to out rule the need for this as a result of potential late-RAD regardless of ORN status/ treatment. The majority of patients (*n* = 8) continued to experience some level of swallowing difficulties at three months post surgery which is in keeping with studies reporting outcomes using validated measures of HRQoL specific to HNC, including domains such as swallowing [2, 12].

In our study, there appeared to be the potential for some deterioration in speech clarity post operatively which appears consistent with previous studies reporting outcomes using the PSS-HN Understandability of Speech subscale [16]. These findings need to be interpreted with caution as both studies included very small samples of 10 patients or less.

Since the data collection period, we are now routinely collecting multidimensional functional data including a range of clinician and patient-reported speech and swallowing measures and instrumental evaluation of swallowing (videofluoroscopy and/ or Flexible Endoscopic Evaluation of Swallowing) for our ORN patients who are being considered for surgical intervention.

## Conclusion

There is limited literature on the nature and extent of speech/ swallowing impairment in patients with advanced ORN prior to and following treatment. Well-designed studies with robust, sensitive multidimensional dysphagia and communication assessments are required. At our centre, we continue to collect prospective multidimensional functional outcome data including routine instrumental evaluation of swallowing, clinician measured, and patient-reported data pre and post surgery for these patients.

## References

[1] Frankart AJ, Frankart MJ, Cervenka B, Tang AL, Krishnan DG, Takiar V (2021). Osteoradionecrosis: Exposing the Evidence Not the Bone. Int J Rad Oncol Biol Phys.

[2] Rogers SN, D'Souza JJ, Lowe D, Kanatas A (2015). Longitudinal evaluation of health-related quality of life after osteoradionecrosis of the mandible. Br J Oral Maxillofac Surg.

[3] Shaw R, Tesfaye B, Bickerstaff M, Silcocks P, Butterworth C (2017). Refining the definition of mandibular osteoradionecrosis in clinical trials: the cancer research UK HOPON trial (Hyperbaric Oxygen for the Prevention of Osteoradionecrosis). Oral Oncol.

[4] Notani KI, Yamazaki Y, Kitada H, Sakakibara N, Fukuda H, Omori K, Nakamura M (2003). Management of mandibular osteoradionecrosis corresponding to the severity of osteoradionecrosis and the method of radiotherapy. Head & Neck: Journal for the Sciences and Specialties of the Head and Neck.

[5] Moon DH, Moon SH, Wang K, Weissler MC, Hackman TG, Zanation AM, Thorp BD, Patel SN, Zevallos JP, Marks LB, Chera BS (2017). Incidence of, and risk factors for, mandibular osteoradionecrosis in patients with oral cavity and oropharynx cancers. Oral Oncol.

[6] Owosho AA, Tsai CJ, Lee RS, Freymiller H, Kadempour A, Varthis S, Sax AZ, Rosen EB, Yom SK, Randazzo J, Drill E (2017). The prevalence and risk factors associated with osteoradionecrosis of the jaw in oral and oropharyngeal cancer patients treated with intensity-modulated radiation therapy (IMRT): the memorial sloan kettering cancer center experience. Oral Oncol.

[7] Aarup-Kristensen S, Hansen CR, Forner L, Brink C, Eriksen JG, Johansen J (2019). Osteoradionecrosis of the mandible after radiotherapy for head and neck cancer: risk factors and dose-volume correlations. Acta Oncol.

[8] Caparrotti F, Huang SH, Lu L, Bratman SV, Ringash J, Bayley A, Cho J, Giuliani M, Kim J, Waldron J, Hansen A (2017). Osteoradionecrosis of the mandible in patients with oropharyngeal carcinoma treated with intensity-modulated radiotherapy. Cancer.

[9] Roe JW, Drinnan MJ, Carding PN, Harrington KJ, Nutting CM (2014). Patient-reported outcomes following parotid-sparing intensity-modulated radiotherapy for head and neck cancer. How important is dysphagia?. Oral Oncol.

[10] Patterson JM, McColl E, Carding PN, Wilson JA (2018). Swallowing beyond six years post (chemo) radiotherapy for head and neck cancer; a cohort study. Oral Oncol.

[11] Hutcheson KA, Lewin JS, Barringer DA, Lisec A, Gunn GB, Moore MW, Holsinger FC (2012). Late dysphagia after radiotherapy-based treatment of head and neck cancer. Cancer.

[12] Wong AT, Lai SY, Gunn GB, Beadle BM, Fuller CD, Barrow MP, Hofstede TM, Chambers MS, Sturgis EM, Mohamed ASR, Lewin JS (2017). Symptom burden and dysphagia associated with osteoradionecrosis in long-term oropharynx cancer survivors: a cohort analysis. Oral Oncol.

[13] Chieng CY, Davies A, Aziz A, Lowe D, Rogers SN (2021). Health related quality of life and patient concerns in patients with osteoradionecrosis. Br J Oral Maxillofac Surg.

[14] Yang D, Zhou F, Fu X, Hou J, Lin L, Huang Q, Yeh CH (2019). Symptom distress and interference among cancer patients with osteoradionecrosis of jaw: A cross-sectional study. Int J Nursing Sci.

[15] Danielsson D, Munck-Wikland E, Hagel E, Halle M (2019). Quality of life after microvascular mandibular reconstruction for osteoradionecrosis—A prospective study. Head Neck.

[16] Shan XF, Li RH, Lu XG, Cai ZG, Zhang J, Zhang JG (2015). Fibular free flap reconstruction for the management of advanced bilateral mandibular osteoradionecrosis. J Craniofacial Surg.

[17] Chandarana SP, Chanowski EJ, Casper KA, Wolf GT, Bradford CR, Worden FP, Eisbruch A, Chepeha DB (2013). Osteocutaneous free tissue transplantation for mandibular osteoradionecrosis. J Reconstr Microsurg.

[18] List MA, Ritter-Sterr C, Lansky SB (1990). A performance status scale for head and neck cancer patients. Cancer.

[19] Kerawala CJ, Riva F, Paleri V (2021). The impact of early oral feeding following head and neck free flap reconstruction on complications and length of stay. Oral Oncol.

[20] Rosenbek JC, Robbins JA, Roecker EB, Coyle JL, Wood JL (1996). A penetration-aspiration scale. Dysphagia.

